# Isolation, identification, and characterization of an *Aspergillus niger* bioflocculant-producing strain using potato starch wastewater as nutrilite and its application

**DOI:** 10.1371/journal.pone.0190236

**Published:** 2018-01-05

**Authors:** Shengyan Pu, Hui Ma, Daili Deng, Shengyang Xue, Rongxin Zhu, Yan Zhou, Xingying Xiong

**Affiliations:** 1 State Key Laboratory of Geohazard Prevention and Geoenvironment Protection (Chengdu University of Technology), Chengdu, Sichuan, P.R. China; 2 Department of Civil and Environment Engineering, The Hong Kong Polytechnic University, Hong Kong, P.R. China; 3 Department of Civil and Environment Engineering, Louisiana State University, Baton Rouge, Louisiana, United States of America; Tallinn University of Technology, ESTONIA

## Abstract

A bioflocculant (MBFA18) was produced by *Aspergillus niger* (A18) using potato starch wastewater (PSW) as nutrients. The cultivation processes and flocculating treatment for PSW purification were systematically studied. The flocculating rate of the MBFA 18 achieved 90.06% (kaolin clay) under the optimal cultivation condition (PSW with 5950 mg/L COD, 20 g/L glucose, 0.2 g/L urea and without phosphorus source addition and pH adjustment). Furthermore, effects of flocculant dosage, initial pH, coagulant aid (CaCl_2_) addition and sedimentation time on the PSW treatment were discussed and studied in detail. The optimum flocculation treatment conditions were determined according to the treatment efficiency, cost and flocculation conditions. During the PSW treatment, 2 mL/L bioflocculant (1.89 g/L) dosage and 0.5 mol/L coagulant aid addition were applied without pH adjustment and 91.15% COD and 60.22% turbidity removal rate could be achieved within 20 min. The comparative study between the bioflocculant and conventional chemical flocculants showed excellent flocculating efficiency of MBFA 18 with lower cost (4.7 yuan/t), which indicated that the bioflocculant MBFA 18 produced in PSW substrate has a great potential to be an alternative flocculant in PSW treatment.

## Introduction

China is the biggest producer of potato starch which accounts for more than 85.9% of the world annual production, thus the domestic potato starch wastewater (PSW) has been becoming a serious problem that needs to be settledd urgently [1]. Two primary issues of PSW, high biological oxygen demand (BOD) and large output amount, bring huge challenges to the traditional treatment technology [2–4]. Suspended substances and chemical oxygen demand (COD) contained in PSW are much higher than in other kinds of wastewater [5]. As an effective and in-situ purification strategy, flocculation technology has drawn much attention for the removal of suspended substances such as solids, colloids, and cell debris during PSW treatment [6]. Flocculants can be classified as inorganic flocculants, like salts of multivalent metals, organic synthetic flocculants such as polyacrylamide and naturally occurring flocculants based on natural polymers or polysaccharides like starch, cellulose, chitosan, natural gums, and mucilage, etc. [7, 8]. Although many inorganic and organic synthetic flocculants[9] have shown good flocculation property, environmental and health problems caused by these flocculants cannot be ignored [10]. Considering the carcinogenicity and biological incompatibility of the conventional inorganic and organic flocculants, the emerging bioflocculant attracts great interest currently because they are harmless, biodegradable and without the problem of producing secondary pollution to the environment [11]. Microbial bioflocculant (MBF), produced by microorganisms during their growth and cell lysis, is an environmental friendly material which can be utilized in water desalination [12], decoloration [13] and in the treatment of drinking water [14], sugarcane wastewater [15] and river water [16]. The *Bacillus mucilaginosus* [17] *Serratia ficaria* [18] and *Aspergillus niger* [19] have been proved as effective bioflocculant producing microorganism[6]. More than 50 kinds of microorganisms ranging from prokaryotic to eukaryotic species have been screened to produce extracellular bioflocculants [20].

However, the high cultivation cost greatly limits the industrialized production and practical application of MBF [21]. Recently, attempts have been made to screen for novel effective bioflocculant-producing microorganisms and seek for economical cultivation substrates [22]. Some mediums such as soybean juice [23] and fishmeal wastewater [24] have been reported as low-cost alternates for the cultivation of flocculant-producing microorganisms[25]. PSW rich in starch, protein, carbohydrates and other organic compounds can be served as a favorable substrate for microbial growth [6]. Therefore, using PSW as a cultivation substrate could be a cost-effective solution for wastewater treatment. Several researches have reported the feasibility of using PSW as a cultivation medium [26].

In this study, a bioflocculant-producing microorganism *Aspergillus niger* (A18) was isolated from soil and cultured in potato starch wastewater substrate. The cultivation and flocculation conditions of fungal bioflocculant were optimized. Furthermore, comparison experiments between conventional chemical flocculants and the resultant bioflocculant were conducted to discuss and study the superiority of different kind of flocculants.

## Methods

### Materials

#### Microorganisms

The bioflocculant-producing microorganism was isolated from 10 cm beneath the soil surface in the Garden of Chengdu University of Technology, Sichuan, China. 1.0 g soil sample was added into 250 mL conical flask with 99 mL sterilized water and glass beads, thoroughly stirring for 20 min to obtain 10^−2^ g/mL soil suspension. Then the soil suspension was diluted to 10^−3^~10^−6^ g/mL. The concentration of 10^−4^, 10^−5^ and 10^−6^ g/mL diluted solution were smeared on the PDA substrate and cultivated upside down under 37°C for 2~3 days. Furthermore, the single colony was separated and inoculated on plate medium cultivated for another 2~3 days. Repeating the separation and purification process until the single colony was obtained. The pure colony was inoculated on the test tube slant preserved at 4°C.

#### Substrate preparation

200.0 g of potato was peeled, minced and boiled in 1000 mL ultrapure water for 30 min with the addition of 20.0 g glucose and 16.0 g agar, without pH adjustment (initial pH 6.38). Then, the mixture was sterilized at 121°C for 20 min to obtain the PDA cultivation medium as isolation substrate. The screening medium was prepared under the same processes above without agar addition.

#### Potato starch wastewater

PSW applied in this study was prepared in laboratory based on the real wastewater quality (Table 1). Potato was peeled and minced to obtain residue, then washed with sterile water at a solid-to-liquid ratio of 1:6 (g:L). Through gauze filtering, the obtained filtrate was standing still for 1 h, and the resultant supernatant was served as PSW.

**Table 1:** Potao starch wastewater quality.

COD_Cr_(mg/L)	TN(mg/L)	TP(mg/L)	pH	Turbidity(NTU)	Chroma(times)
10130	604.6	84.3	6.30	467	128

### Experimental details

#### Screening and identification of bioflocculant-producing microorganism

Flocculant producing-microorganisms were isolated twice to select the microorganisms with high flocculation capability. (1) Preliminary screening: 2.5 mL of each diluted sample (5% v/v) was inoculated into 50 mL PDA substrate and cultivated in a rotary shaker under 28°C at 150 rpm for 2 days. Several drops of fermentation broth were added into 100 mL kaolin suspension (5 g/L). The turbidity of the suspension was measured to determine the flocculating capability of each microorganism. Six bioflocculant-producing microorganisms with flocculating efficiency higher than 80% were selected for the following research. (2) Secondary screening: The secondary screening was conducted in the same way, and the microorganism with the highest flocculating efficiency was isolated as the ultimate bioflocculant producer.

The colony and hypha morphologies of the resultant bioflocculant-producing microorganism were observed using microscope and identified according to *Environmental Microorganism Identification* [27].

#### Preparation of spore suspension

20 mL sterile water was added to the fungal cultivation slant. The slant was scraped by an inoculating loop and stirred in a sterile conical flask with glass beads and sterile water for 1 min. Then, the concentration of the spore was adjusted to ca. 10^7^ unit/L and stirred thoroughly at 150 r/min under 28°C for 2 days to obtain spore suspension.

#### Distribution of the flocculating activity

The fermentation broth was centrifuged at 4000 r/min for 10 min to separate mycelia and liquid supernatant. To determine the distribution of the flocculating activity, flocculating rate of the same volume of liquid supernatant of the fermentation broth, mycelial suspension without washing, mycelial suspension after washing and fermentation broth contain mycelia without centrifugal separation were measured to determine the distribution of the flocculating capacity. 0.5 g kaolin clay with diameter around 10 μm was added into 100 mL deionized water to prepare kaolin suspension. 0.1 mL of different bioflocculant was added in the suspension and stirred thoroughly (400 r/min for 0.5 min, 120 r/min for 2 min and 80 r/min for 10 min, respectively). The mixture was settled for 10 min, then the turbidity of liquid (1 cm beneath the fluid level) was measured. The turbidity of kaolin suspension given the same treatment without bioflocculant addition was measured as blank control. The flocculating rate was calculated as follow:

Flocculatingrate(%)=(A−B)/A×100%

where A and B are the turbidity of the blank control and the suspension treated by bioflocculant respectively.

#### Purification of the bioflocculant

The fermentation broth was centrifuged at 4000 r/min for 10 min, then the supernatant was placed into cold ethanol centrifuged at 4000 r/min for another 10 min. The obtained precipitate was dried under 60°C to get the crude bioflocculant.

#### Bioflocculant composition and thermal stability analysis

The flocculating rate of the fermentation supernatant heated under different temperature for a different period of time was measured to determine the thermal stability of the bioflocculant [28]. The composition of the bioflocculant was analyzed by the reactions below:

Saccharide determination. Molish reaction: Furfural and its derivatives were synthesized by a dehydration reaction between carbohydrate with concentrated sulfuric acid, and then the resultant reacts with alpha-naphthol to form amaranth compound.

Anthrone reaction: Anthrone reacts with saccharides producing cyan compound under the treatment of concentrated sulfuric acid.

Protein and amino acid determination. Ninhydrin reaction: Alpha-amino acid and protein can react with ninhydrin to produce the bluish violet compound, which can be used to determine the existence protein and amino acid.

Biuret reaction: Polypeptide in protein with a similar structure of biuret combined with Cu^2+^ generates amaranth compound under alkaline condition.

Protein yellow reaction: With concentrated nitric acid, the yellow compound can be produced by heating the benzene ring containing protein mixture.

Fourier transform infrared spectroscopy (FT-IR) analysis was used to characterize the functional groups of the prepared bioflocculant.

#### Optimization of wastewater cultivation conditions

The effects of the wastewater sterilization condition, carbon sources category, nutrilite concentration, inoculation amount, initial pH of medium and cultivation time were studied and optimized, and the flocculating rate of kaolin suspension was used to determine the optimal bioflocculant-producing microorganism cultivation condition. The optimization experiments were carried out in 50 mL wastewater substrate. After adjusting the cultivation condition, the flakes were put in a rotary shaker at 150 r/min, 28°C for 2 days.

#### PSW treatment using the novel bioflocculant

The novel bioflocculant was produced under the optimal culture condition. During the wastewater treatment, the effects of bioflocculant dosage, type, and the addition of coagulant, initial pH and sedimentation time were systematically investigated and optimized to enhance the flocculating rate.

### Comparative study of chemical flocculant and bioflocculant in PSW treatment

The flocculating behavior of two most common chemical flocculants Al_2_(SO_4_)_3_ and FeCl_3_ were investigated as a comparison. The effects of the type and dosage of flocculant and coagulate, initial pH and settling time on the flocculating efficiency were investigated. Meanwhile, the flocculating effects and cost of the chemical flocculant and bioflocculant were discussed in detail under optimal treating conditions.

### Instruments

HNY-100D gas bath thermostatic oscillator; KXH-25A biochemical incubator; YX280A Portable stainless steel steam sterilizer; 85–2 Digital display temperature control magnetic stirrer; B203LED biological microscope; DHG-9240B constant temperature oven; TDL-4A table centrifuge.

## Results and discussion

### Bioflocculant production

#### Screening and identification of microorganisms

After extraction, 20 strains of fungi including 17 strains of microzyme and 11 strains of mould were isolated from soil samples. The fermentation broth of these fungi was added into kaolin suspension. The preliminary screening results of the 6 strains of the fungi isolated from soil samples with high flocculating efficiency were shown in Table 2. To select the optimum strains, the further screening experiment about these 6 strains was conducted to exclude uncertain factors.

**Table 2:** Results of preliminary and further screening.

**Preliminary Screening**	Strain number	J3	J6	J14	J25	M18	M19
	Flocculating rate (%)	81.43	85.47	81.74	93.12	94.44	90.12
**Further Screening**	Strain number	J3	J6	J14	J25	M18	M19
	Flocculating rate (%)	82.35	87.54	79.77	92.34	95.26	89.50

The screening results revealed that the fermentation broth of fungi M18 had the highest flocculation rate.

The spore area of M18 on PDA substrate presented a black and powder morphology. The double layer peduncle and dandelion-like acrocyst were easily found under microscope and the M18 were identified as *Aspergillus niger* (A18), shown in Supporting Information (S1 Fig). The fermentation broth generated was designated as bioflocculant MBFA18.

#### Distribution of flocculating activity and purification of the bioflocculant

**Fig 1:**
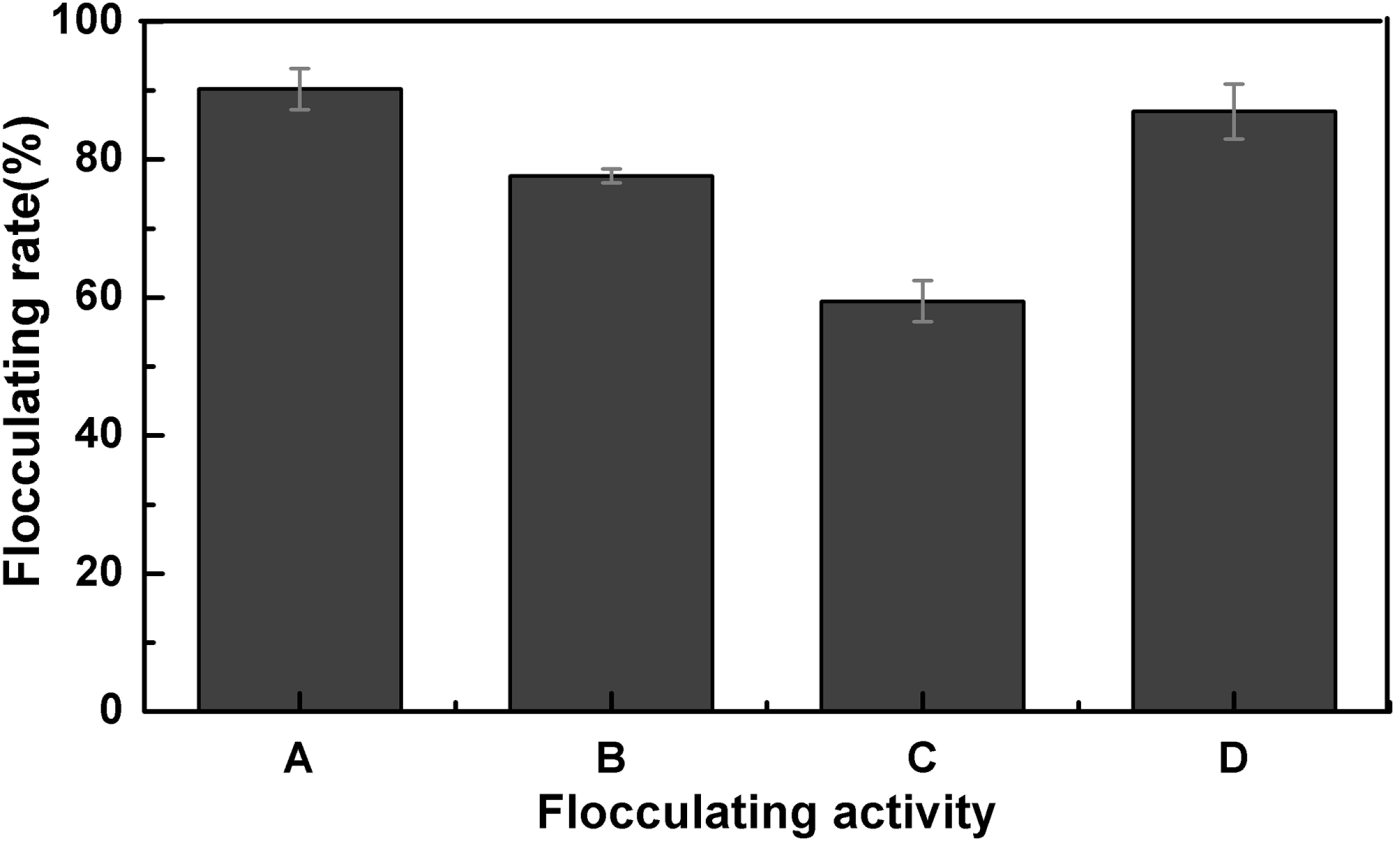
Flocculating activity distribution of the microorganism and its fermentation broth. A: Fermentation broth supernatant; B: Mycelial pellets without washing; C:Washed mycelial pellets; D: Fermentation broth.

The hyphae of A18 convolved to white mycelial pellets in clear fermentation broth during shaking in PDA medium. The flocculating rate of each part of the fermentation broth was shown in Fig 1. The supernatant showed the highest flocculating rate, which indicated the flocculating substance secreted by fungal mainly distributed in the supernatant. The white mycelial pellets before and after washing presented flocculating activity due to their adsorption ability. In addition, the existence of the fungi did not affect the flocculation process.

For the purification of the flocculant, 120 mL fermentation broth was precipitated with 240 mL cold ethanol. After centrifugation and drying, 0.2267 g of colorless and transparent crystal was obtained as crude bioflocculant.

#### Thermal stability and composition analysis of MFB18

**Fig 2:**
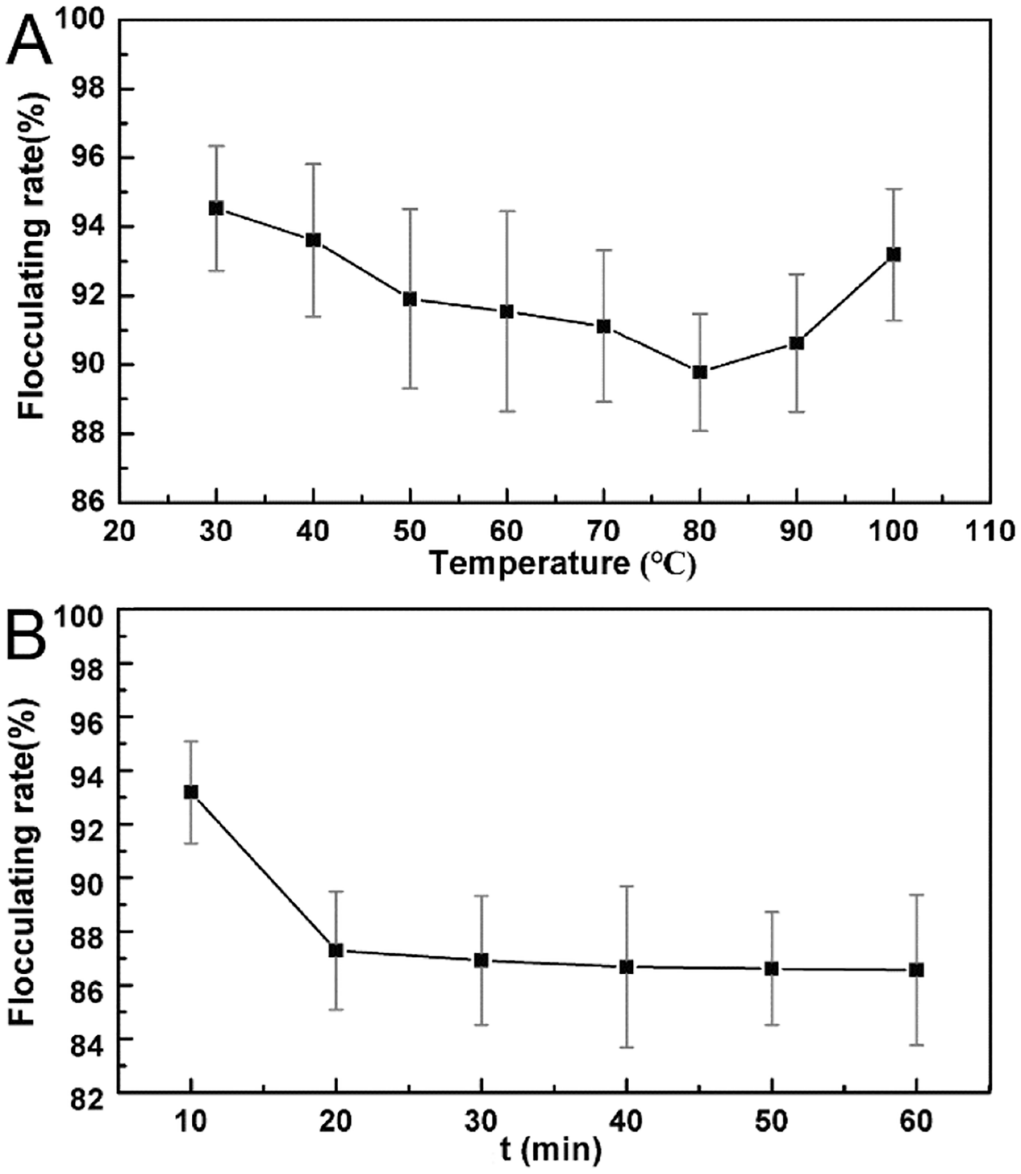
A: Effect of heating temperature on flocculating activity; B: Effect of heating time on the flocculating activity.

As shown in Fig 2A, after boiling for 10 min, the flocculation rate decreased slightly with the temperature, then presented an increasing tendency after 80°C. Nevertheless, the flocculating rates were higher than 89%, and the flocculating ability of MFBA 18 was not affected severely by heating. The flocculating rate decreased less than 6% after heating at 100°C for 60 min (Fig 2B), which indicated the excellent thermal stability of the obtained bioflocculant.

Chromogenic reactions were used to analyze the composition of MFB18 bioflocculant. Combining the results of thermal stability test (Fig 2) and the chromogenic reaction results (Table 3), the main component of MFB18 was evaluated as polysaccharide instead of protein which denatured easily in boiling water.

**Table 3:** Composition analysis by chromogenic reactions.

	Experiment item	Experiment phenomena	Experimental conclusion
1	Molish reaction	Clear purple ring was found on the interface of concentrated sulfuric acid and sample (S2 Fig)	carbohydrate
2	Anthrone reaction	The liquid presented cyan (S3 Fig)	Polysaccharide
3	Biuret reaction	The color of liquid did not change	Without polypeptide
4	Ninhydrin reaction	There was no phenomenon	Without protein
5	Protein yellow reaction	It did not present any change	Without protein

**Fig 3:**
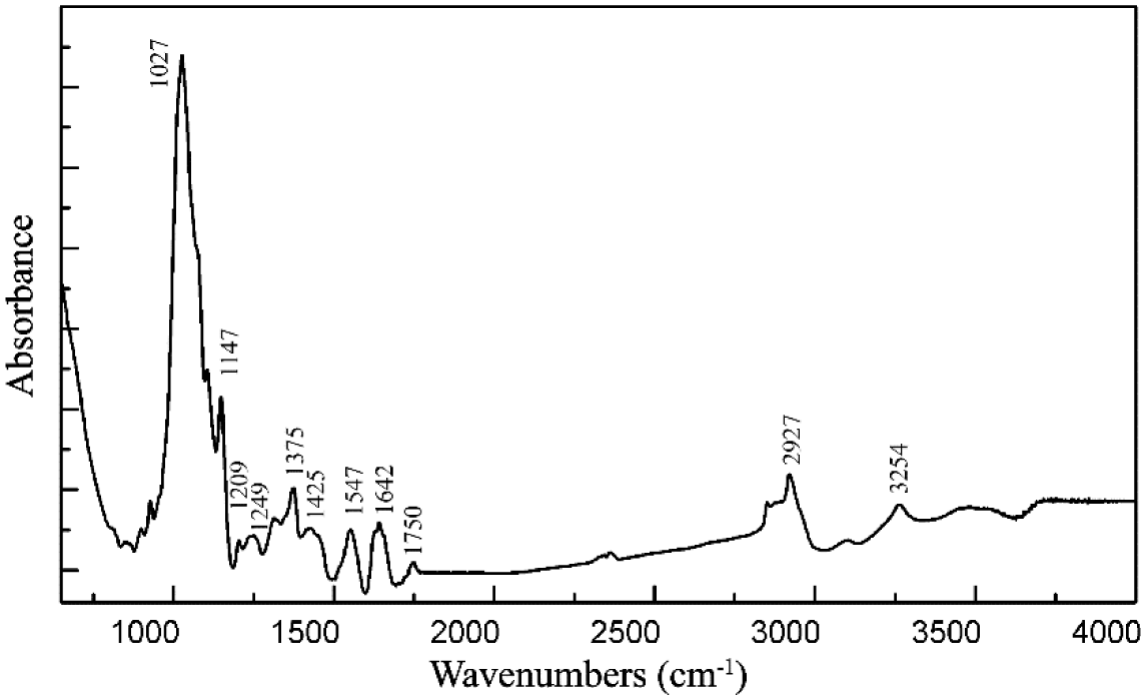
FT-IR spectrum of the bioflocculant MFB18.

FT-IR was applied to analyze the functional groups of the bioflocculant. As shown in Fig 3, the -OH stretching and weak C-H stretching vibration band was observed at 3254 cm^-1^ and 2927 cm^-1^, respectively. The peak at 1750 cm^-1^ was characteristic of the C = O stretching vibration in -COOH [17]. The asymmetrical stretching band at 1642 cm^-1^ confirmed the presence of carboxylates. The peak at 1425 cm^-1^ indicated the presence of carboxylic acid group and polysaccharide, and the peak at 1249 cm^-1^ was an indication of the C-O stretching. The peaks at 1027 cm^-1^, 1147 cm^-1^, and 1209 cm^-1^ were characteristic of all sugar moieties[29].

### Optimization of wastewater cultivation conditions for MFB18

#### Effect of sterilization on flocculating activity

A large number of microorganisms were contained in potato starch wastewater. To investigate the influence of the indigenous microorganisms on the MFB18 flocculating ability, the wastewater sterilization condition was studied. 50 mL PSW substrate was sterilized at 121°C and kept at room temperature (25°C). 2.5 mL M18 spore suspension was inoculated into the sterilized and unsterilized substrates respectively, cultivated at 150 r/min under 28°C for 2 days. Moreover, the flocculating rates of the fermentation broth from each sample were measured. The fermentation broth produced in sterilized substrate showed higher flocculating rate (70.81%) than the unsterilized one (62.11%), which indicated that there was no microorganism symbiosis or synergistic effect in the flocculating process. Meanwhile, it should be noted that sterilization only led an increase less than 10% enhancement in flocculating efficiency. Considering the industrial reality and the cost of the application, wastewater would not be sterilized in the following research.

#### Effect of addition of carbon source on flocculating activity

**Fig 4:**
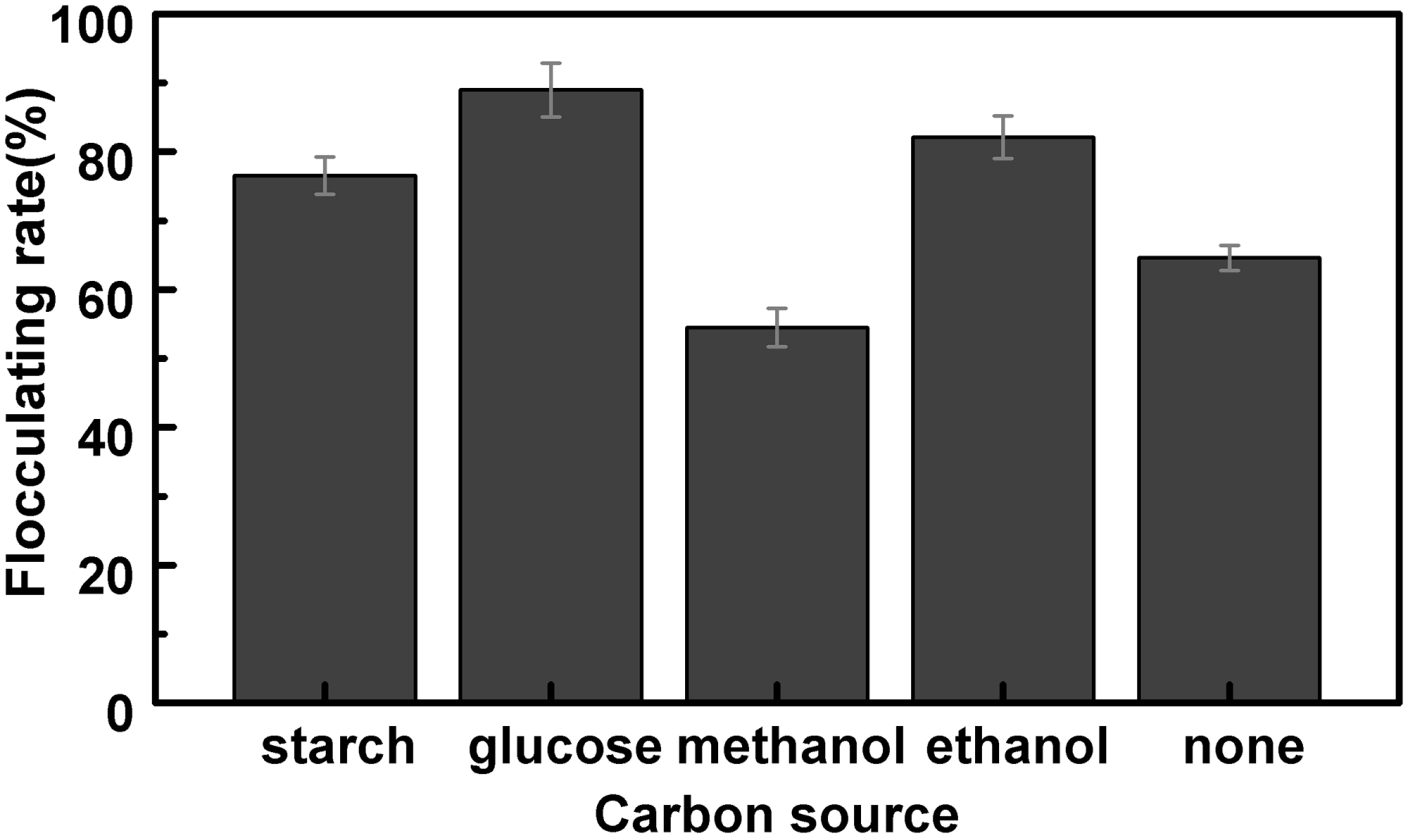
Effect of carbon source on flocculating activity.

The carbon source in the substrate was an essential factor for the microorganism cultivation, which affected the flocculating activity indirectly. 1g starch, 1g glucose, 1 mL ethanol and 1 mL methanol were added into 4 conical flasks containing 50 mL potato starch wastewater substrate, respectively. 5% (v/v) of the fungi was inoculated in the medium cultivated under 28°C at 150 r/min for two days. The blank control sample was prepared under the same condition without carbon source addition. The flocculating rates of the fermentation broths were shown in Fig 4.

Compared with blank control, flocculating rates of the bioflocculant increased due to the addition of starch, glucose and ethanol, while the addition of methanol caused inhibition effect. Glucose promoted the flocculating process to the maximal extent (88.98%) among others by accelerating the bacteria growth. Therefore, glucose was selected as the carbon source in the following experiments.

#### Effect of nutrilite addition on flocculating activity

The orthogonal array experiment of four factors and four levels was designed to analyze the effects of COD concentration (factor A) of PSW, the concentration of carbon (factor B: glucose), nitrogen (factor C: 10 g/L urea) and phosphorus (factor D:10 g/L potassium dihydrogen phosphate) sources on the flocculating efficiency. The importance of each factor for the bioflocculant was different. The range analysis was used to select the optimal condition. ${\bar{\text{K}}}_{\text{jm}}$ is defined as the average of the evaluation indexes of all levels (m = 1, 2, 3, 4) in each factor, which is used to determine the optimal level and the optimal combination of factors. The optimal level for each factor could be obtained when ${\bar{\text{K}}}_{\text{jm}}$ is the largest. R_j_ is defined as the range between the maximum and minimum value of ${\bar{\text{K}}}_{\text{jm}}$, which is used for evaluating the importance of the factors (the greater R_j_ means the greater importance of the factor).

$${\bar{K}}_{jm}=\frac{K_{j1}+K_{j2}{+K}_{j3}{+K}_{j4}}{4}$$

$$R_{j}={\bar{K}}_{max}-{\bar{K}}_{min}$$

As shown in Table 4, high COD concentration was unfavorable for the growth of the microorganism due to the high osmotic pressure. In addition, high COD also increased the cultivation cost. According to the R_j_ value, the results showed that the importance of factors for the flocculating rate was: glucose > urea > COD > potassium dihydrogen phosphate (MKP). Glucose addition was the most influential factor for flocculating rates, while the phosphorus source had the least effect. The result of range analysis showed A_2_B_3_C_3_D_1_ (S1 Table) was the optimal cultivation condition, which achieved a flocculating rate of 90.84%. A verification experiment using bioflocculant produced under this condition reached a 92.24% flocculating rate. In summary, the optimal cultivation condition for fermentation broth producing was as followed: 5950 mg/L COD, 20 g/L glucose dosage, 10 g/L urea addition and none addition of potassium dihydrogen phosphate.

**Table 4:** Results of the effect of nutrilite addition on flocculating activity analyzed by orthogonal array experiment.

NO.	Group	COD(mg/L)	Glucose (g)	Urea (mL)	MKP(mL)	Flocculating rate(%)
1	A_1_B_1_C_1_D_1_	10130	0.0	0.0	0.0	60.87
2	A_1_B_2_C_2_D_2_	10130	0.5	0.5	0.2	74.07
3	A_1_B_3_C_3_D_3_	10130	1.0	1.0	0.5	83.85
4	A_1_B_4_C_4_D_4_	10130	1.5	1.5	1.0	88.04
5	A_2_B_1_C_2_D_3_	5950	0.0	0.5	0.5	70.34
6	A_2_B_2_C_1_D_4_	5950	0.5	0.0	1.0	78.73
7	A_2_B_3_C_4_D_1_	5950	1.0	1.5	0.0	90.53
8	A_2_B_4_C_3_D_2_	5950	1.5	1.0	0.2	89.13
9	A_3_B_1_C_3_D_4_	5060	0.0	1.0	1.0	68.32
10	A_3_B_2_C_4_D_3_	5060	0.5	1.5	0.5	77.64
11	A_3_B_3_C_1_D_2_	5060	1.0	0.0	0.2	88.35
12	A_3_B_4_C_2_D_1_	5060	1.5	0.5	0.0	89.90
13	A_4_B_1_C_4_D_2_	3560	0.0	1.5	0.2	63.82
14	A_4_B_2_C_3_D_1_	3560	0.5	1.0	0.0	90.84
15	A_4_B_3_C_2_D_4_	3560	1.0	0.5	1.0	89.90
16	A_4_B_4_C_1_D_3_	3560	1.5	0.0	0.5	80.12
${\bar{\text{K}}}_{j1}$		76.71	65.84	77.02	83.04	
${\bar{\text{K}}}_{j2}$		82.18	80.32	81.05	78.84	
${\bar{\text{K}}}_{j3}$		81.05	88.16	83.04	78.00	
${\bar{\text{K}}}_{j4}$		81.17	86.80	80.01	81.25	
R_j_		5.47	22.35	6.02	5.04	

#### Effect of initial pH on flocculating activity

**Fig 5:**
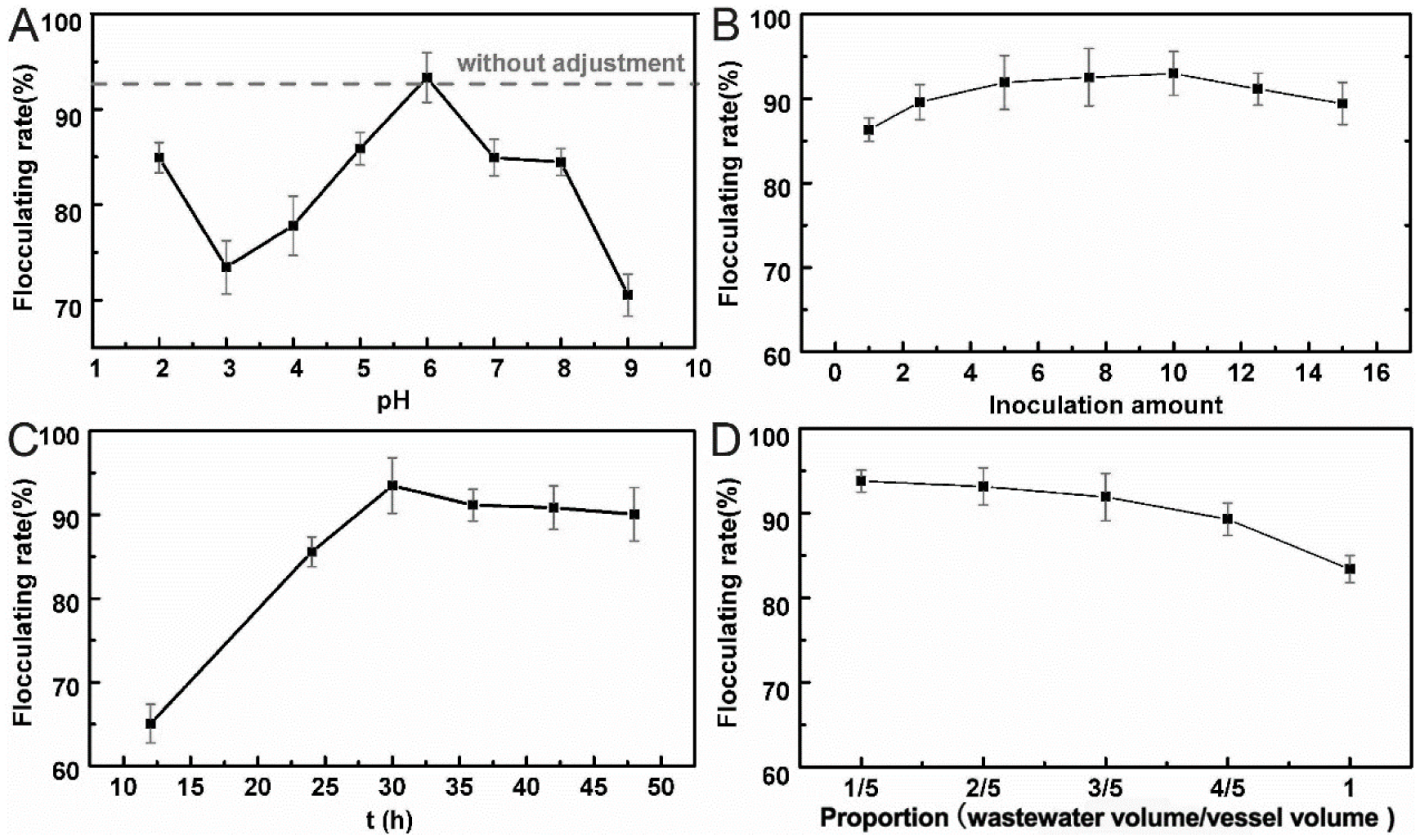
A: Effect of pH on flocculating activity; B: Effect of inoculation amount on flocculating activity; C: Effect of culturing time on flocculating activity; D: Effect of the proportion of wastewater medium volume and vessel cubage on the flocculating.

The initial pH of the substrate was another important factor, which affected the growth and metabolism of the microorganism. M18 was inoculated into 50 mL wastewater cultivation substrate at different initial pH values under optimal cultivation conditions. The flocculating activity was measured and analyzed by the flocculating efficiency. As shown in Fig 5A, the fermentation broth presented high flocculating efficiency (above 84%) in the initial pH ranging from 5 to 8. The highest flocculating rate 93.32% was achieved at pH 6, which was the same initial pH without adjustment. Therefore, no pH adjustment was applied in the following experiment.

#### Effect of total inoculation amount and cultivation time on flocculating activity

Different amounts of fungal suspension (10^7^ unit/mL) was inoculated into the cultivation substrate to achieve a final inoculation proportion of 1%, 2.5%, 5%, 7.5%, 10%, 12.5% and 15% (v/v), respectively. Fermentation broth produced by M18 was added into 100 mL kaolin suspension. The flocculating rate reached the highest (93.1%) when the inoculation amount was 10% as shown in Fig 5B. When the inoculation was relatively low, microorganism grew freely, thus flocculating rate increased with inoculation amount. However, growth competition limited the production of the bioflocculant particularly when inoculation amount was higher than 10%.

Different growth status of M18 led to distinct flocculating performances, which affected by the microorganism cultivation time. Fermentation broth produced by microorganism in different growth stages needed different dosage to start flocculation as shown in Table 5, and the flocculants were added into kaolin suspension until the flocs emerging. Generally, the period of microorganism growth could be divided into lag, logarithmic, stationary and decay phase. The results shown in Fig 5C indicated that, during the first 12 h, the microorganism produced a low amount of active substance due to the adaption period in the adjustment phase. Then microorganism entered into the logarithmic growth phase in 12~30 h, where active growth and microorganism metabolism occurred and the flocculating efficiency increased. And in 30~48 h, A18 entered into stable phase.

**Table 5:** Dosage of bioflocculant cultured different dosage and time.

Time (h)	12	24	30	36	42	48
Dosage (mL)	5	0.4	0.2	0.2	0.2	0.2
Flocculating rate (%)	65.06	85.56	93.48	91.15	90.84	90.06

#### Effect of proportion of wastewater medium volume and vessel cubage on the flocculating rate

The fungi M18 used in this study was an aerobic microorganism, which needs enough oxygen for its growth and metabolism. The proportion of the wastewater volume changed the space and the shear force in the conical flask, which had a great effect on the dissolved oxygen concentration of the substrate. 250 mL conical flasks containing a different volume of wastewater were used to culture M18 and the flocculating efficiency of the produced fermentation broth for kaolin suspension was measured. As shown in Fig 5D, when the air volume was less than 1/5 of the flask volume, the flocculating rate of the produced fermentation broth decreased sharply due to poor-oxygen cultivation condition. When the proportion of substrate volume less than 4/5, the activity of the bioflocculant was not affected severely. In conclusion, the vessel should have at least 1/5 of air to ensure the flocculating efficiency of MBFA 18.

### Benefits from the cultivation of M18 using PSW wastewater

The primary purpose of producing bioflocculant cultivated by PSW substrate was to recycle waste resource and reduce processing cost. The COD concentration of the potato starch wastewater after cultivating M18 was 980 mg/L with a 93.60% removal rate and the turbidity is 56 NTU with an 82.87% removal efficiency. Using PSW as nutrition cut the cost significantly without loss of yield and flocculating efficiency of the bioflocculant (Table 6). After the flocculation treatment, the effluent could be processed easily or irrigate potato plant field directly after mixing with fresh water.

**Table 6:** Comparison of microorganisms cultivated in PDA and PSW substrate.

Culture substrate	Cost of substrate (yuan/L)	Bioflocculant yield (g/L)	Optimal dosage (mL/L)	Flocculating rate (%)
PDA	2.34	1.889g/l	1	95.26%
PSW	0.646	0.820g/l	2	90.06%

### PSW treatment using bioflocculant

As a low-cost, non-toxic and effective bioflocculant, the effects on processing condition such as addition dosage, initial wastewater pH, species of coagulant addition and settling time were investigated.

**Fig 6:**
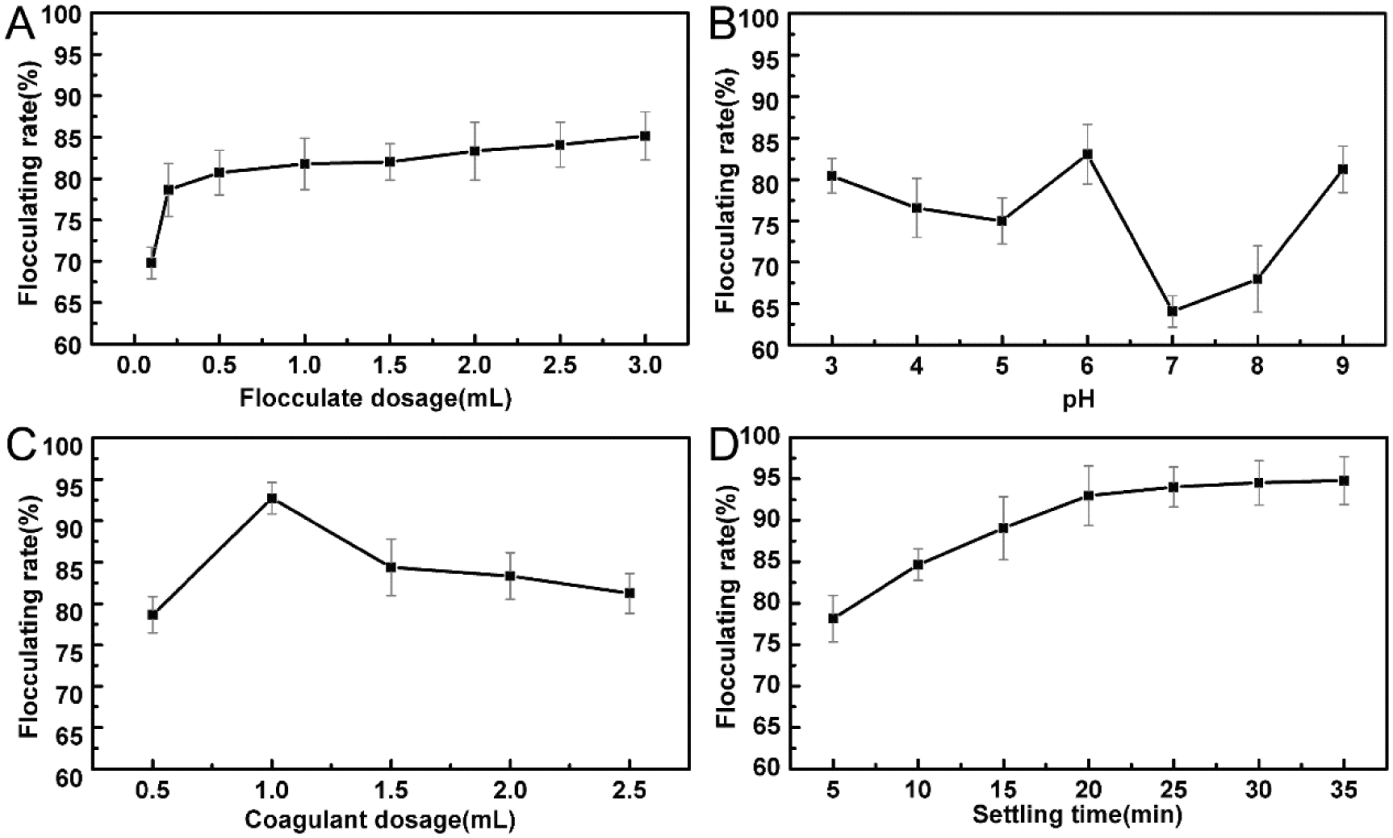
A: Effect of flocculant dosage on flocculating activity; B: Effect of pH on flocculating activity; C: Effect of coagulant dosage on flocculating activity; D: Effect of settling time on flocculating activity.

As shown in Fig 6A, flocculating rate increased with the increase of flocculant dosage. However, electrostatic repulsion between suspended particles caused by large dosage also inhibited the flocculation process significantly. Initial pH was another important factor for the flocculating activity due to its effect on the surface electrical charge of the suspended particles. The flocculating rate reached the maximum at pH 6 shown in Fig 6B, thus pH adjustment was unnecessary during the treatment. The flocculation results of metal cations acted as a coagulant in the flocculation process was shown in Table 7. The flocculating rates were promoted by addition of Ca^2+^, Mg^2+^, Ba^2+^, Al^3+^, Fe^3+^, and restrained by addition of Na^+^ and K^+^.

**Table 7:** Effect of cations on the flocculating rate activity of bioflocculant.

Cations	Na^+^	K^+^	Mg^2+^	Ca^2+^	Ba^2+^	Cu^2+^	Al^3+^	Fe^3+^
**Flocculating rate(%)**	67.44	74.48	85.42	92.97	92.71	78.65	93.23	94.27

Since Al^3+^ and Ba^2+^ cause biological toxicity and Fe^3+^ caused rust in water, Ca^2+^ was selected as an optimal coagulant in the flocculation process due to its high flocculating rates. Moreover, the effect of coagulant dosage and settling time were investigated. The addition of Ca^2+^ had a positive impact on the flocculation due to the formation of adsorption bridging and net catching effect. However, excess Ca^2+^ also occupied a large number of active sites. As shown in Fig 6C, the highest flocculation rate was achieved with the addition of 10 mL/L of CaCl_2_. Initial settlement rate of flocs reached 92.79% within 20 min as shown in Fig 6D, which confirmed the excellent flocculation efficiency of the fermentation broth. Table 8 summarized the bioflocculant-producing microorganisms and the flocculating conditions reported in other recent researches. The comparison illustrated that the potato starch wastewater was an ideal cultivation medium for *Aspergillus niger* to produce the effective bioflocculant MBFA 18.

**Table 8:** Comparison bioflocculant in other research on wastewater treatment.

Flocculant	Source	Dosage (mg/L)	pH	Treated wastewater	Removal efficiency	Ref
MBF-6	*Klebsiella pneumoniae*	50	7	Kaolin suspension(5g/L)	93.87%	[30]
p-KG03	*Gyrodinium impudicum KG03*	1	4	Kaolin suspension(5g/L)	90%	[31]
Bacillus pumilus	*Bacillus pumilus strain ZAP 028*	100	10	Kaolin suspension(4g/L)	95.9%	[32]
pKr	*Kocuria rosea BU22S*	1.2	7	Kaolin suspension(4g/L)	92%	[33]
IH-7	*Aspergillus flavus*	1	7	Kaolin suspension(2g/L)	97.4%	[34]
MBF-C9	*Bacillus agaradhaerens C9*	5	6.53	Kaolin suspension(5g/L)	95.29	[35]
DYU500	*Bacillus subtilis DYU1*	40	6~7	Kaolin suspension(5g/L)	97%	[36]
MBFA18	*Aspergillus niger*	3.778	6	Kaolin suspension(5g/L)	92.97%	This work

### Comparative study of flocculating efficiency of biofocculants and chemical flocculants

**Fig 7:**
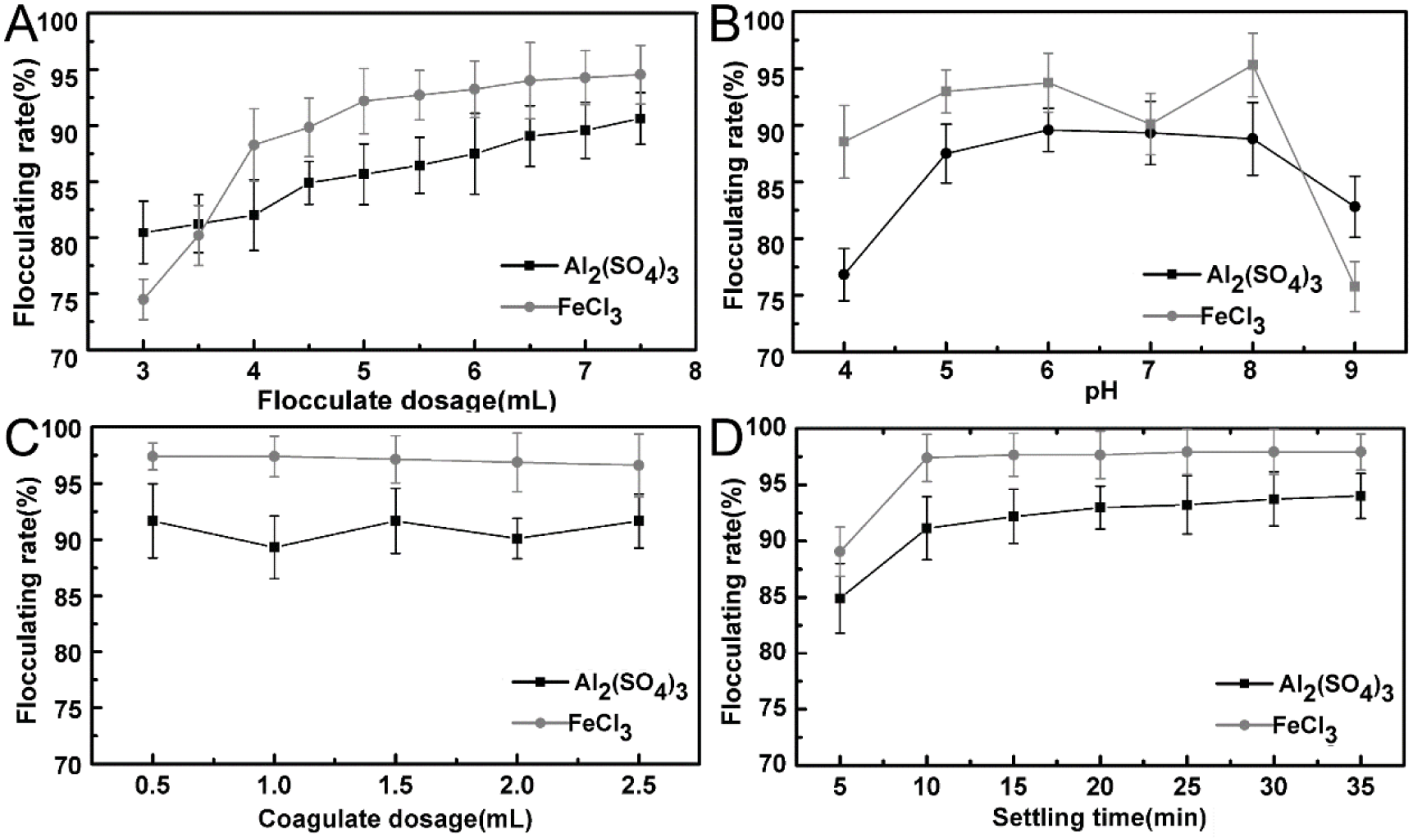
A: Effect of chemical flocculant dosage on flocculating activity; B: Effect of pH on flocculating activity; C: Effect of coagulate dosage on flocculating activity; D: Effect of settling time on the flocculating activity.

The flocculating behavior of two the most typical chemical flocculants, Al_2_(SO_4_)_3_ and FeCl_3,_ were investigated for the comparison study. The influence of flocculant and coagulate dosage, initial pH and settling time on flocculating rate were investigated under the optimal condition as shown in Fig 7. To obtain an acceptable flocculating effect, 7 mL Al_2_(SO_4_)_3_ and FeCl_3_ solution (10 g/L) were determined as optimal flocculant dosage, respectively (Fig 7A). The flocculating rate increased slowly when the concentration of chemical flocculant was higher than 700 mg/L. The addition of coagulate promoted the flocculating process distinctively, yet the impact of the dosage variation on the flocculating system was negligible. Thus the dosage of coagulate was determined as 5 mL/L of CaCl_2_ solution (0.5 mol/L) to cut the processing cost. The flocculating rate of Al_2_(SO_4_)_3_ achieved the highest at pH 6.0 and FeCl_3_ at pH 8.0 (Fig 7B). The settling efficiency with flocculants Al_2_(SO_4_)_3_ and FeCl_3_ increased fast and reached the highest in first 10 min. Therefore, 10 min was determined as the optimal settlement time for Al_2_(SO_4_)_3_ and FeCl_3_ (Fig 7D).

Under optimal condition, the comparison results of chemical flocculants (Al_2_(SO_4_)_3_ and FeCl_3_) and bioflocculant (MBFA 18) were shown in Table 9. The MFB18 maintained higher flocculation efficiency with less dosage, sludge amount and moderate treating condition. The processing cost of the bioflocculant was 4.7 yuan/t, which is much lower than that of chemical flocculants. Moreover, 0.165 g/L of proteic substance was recycled as a byproduct, which could be utilized as animal feed or fertilizer further.

**Table 9:** Comparison of chemical flocculants and bioflocculant on wastewater treatment.

Flocculant	Dosage of flocculant (mg/L)	pH	Dosage of coagulant	Settling time (min)	Amount of sludge(g/L)	Removal rate (%)		Cost (yuan/t)
						Turbidity	COD	
Al_2_(SO_4_)_3_	700	natural	5mL/L	10	0.89	92.97	60.22	32.2
FeCl_3_	700	8	5mL/L	10	0.42	97.66	66.93	24
MBFA18	3.778	natural	10mL/L	20	0.165	92.97	61.01	**4.7**

## Conclusions

This study revealed that PSW could serve as a suitable substrate for the bioflocculant producing microorganism- *Aspergillus niger* (A18) isolated from soil sample. MBFA18 produced by A18 showed excellent flocculating efficiency under the optimal cultivation condition (PSW with the addition of 5950 mg/L COD, 20 g/L glucose dosage, 10 g/L urea and without the addition of phosphorus source and further pH adjustment). In the PSW treatment, the removal efficiency of COD and turbidity by MBFA18 reached 91.15% and 60.22%, respectively. The bioflocculant MBFA 18 with less dosage, sludge amount and moderate treating condition presented a comparable flocculating efficiency to the chemical flocculant in the PSW treatment, and the lower treatment cost the of MBFA 18 improved the feasibility of application prospect of using PSW as a medium for bioflocculant producing.

## Supporting information

S1 Table L16(44) form of orthogonal array experiment for analyzing the effect of nutrilite addition on flocculating activity. (DOCX)

S1 Fig The morphology of the A18. A: *Aspergillus niger* A18 colony morphology figure; B: 40 times magnification of *Aspergillus niger* A18 under microscope; C: Morphology of *Aspergillus niger* A18 fermentation broth and hypha ball.
(TIF)

S2 Fig Molish reaction of the fermentation broth for the component analysis. (TIF)

S3 Fig Anthrone reaction of the fermentation broth for the component analysis. (TIF)
