# Supplementary material for: Isolation, identification, and characterization of an Aspergillus niger bioflocculant-producing strain using potato starch wastewater as nutrilite and its application
Source: PLoS One. 2018 Jan 5;13(1):e0190236. doi: 10.1371/journal.pone.0190236 (PMC5755778; doi:10.1371/journal.pone.0190236)
Supplement: S1 Table — (DOCX) [file pone.0190236.s001.docx]

**S1 Table. L16(44) form of orthogonal array experiment for analyzing the effect of nutrilite addition on flocculating activity.**

| Factor  Level | A(mg/L) | B(g) | C(mL) | D(mL) |
| --- | --- | --- | --- | --- |
| 1 | 10130 | 0.0 | 0.0 | 0.0 |
| 2 | 5950 | 0.5 | 0.5 | 0.2 |
| 3 | 5060 | 1.0 | 1.0 | 0.5 |
| 4 | 3560 | 1.5 | 1.5 | 1.0 |
