# Supplementary figures and images for: Isolation, identification, and characterization of an Aspergillus niger bioflocculant-producing strain using potato starch wastewater as nutrilite and its application
Source: PLoS One. 2018 Jan 5;13(1):e0190236. doi: 10.1371/journal.pone.0190236 (PMC5755778; doi:10.1371/journal.pone.0190236)

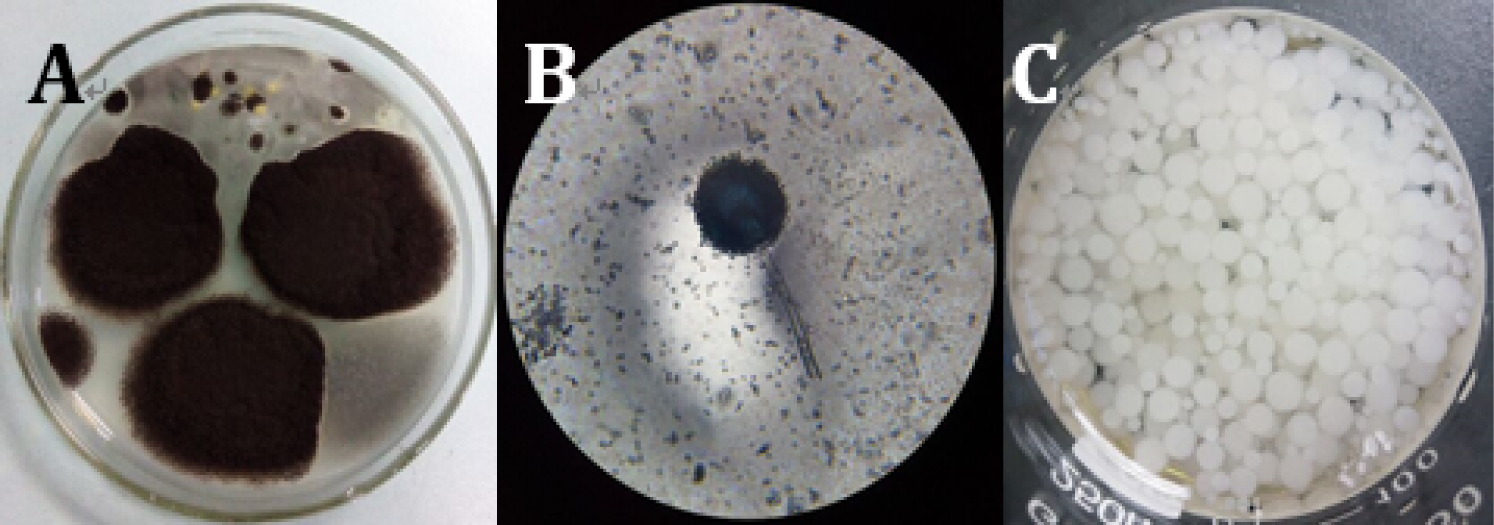

Supplement: S1 Fig — A: Aspergillus niger A18 colony morphology figure; B: 40 times magnification of Aspergillus niger A18 under microscope; C: Morphology of Aspergillus niger A18 fermentation broth and hypha ball. (TIF) [file pone.0190236.s002.tif]

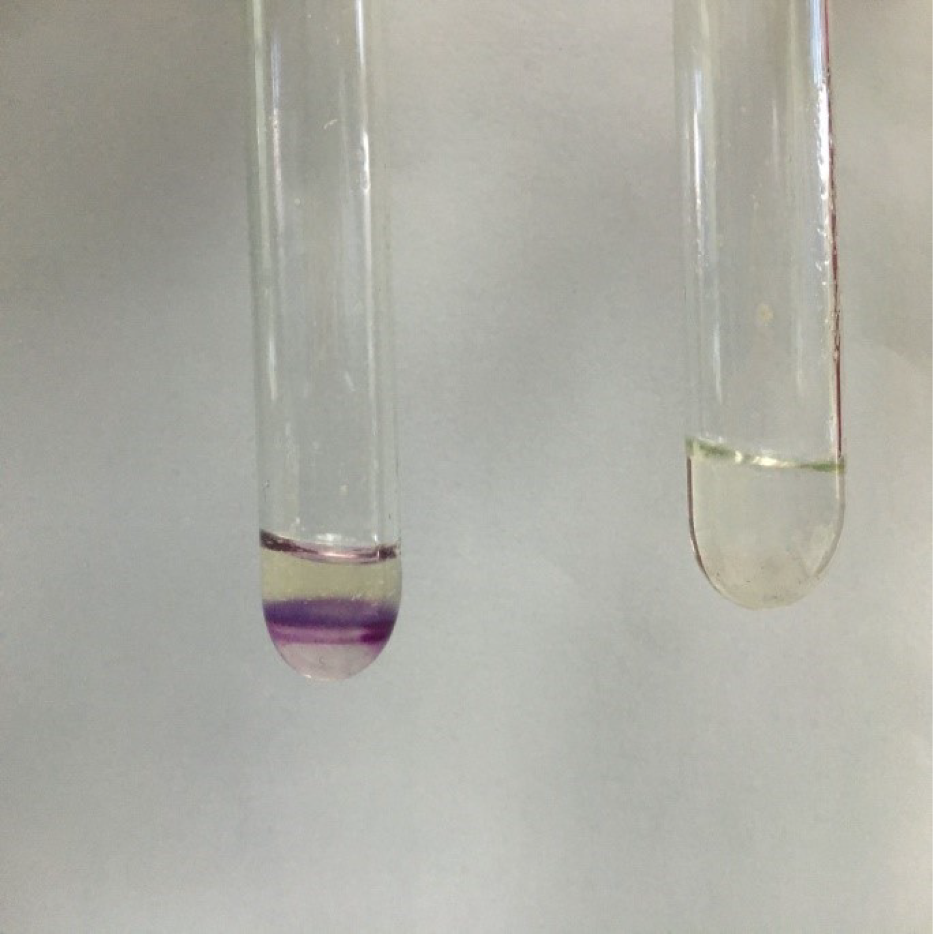

Supplement: S2 Fig — (TIF) [file pone.0190236.s003.tif]

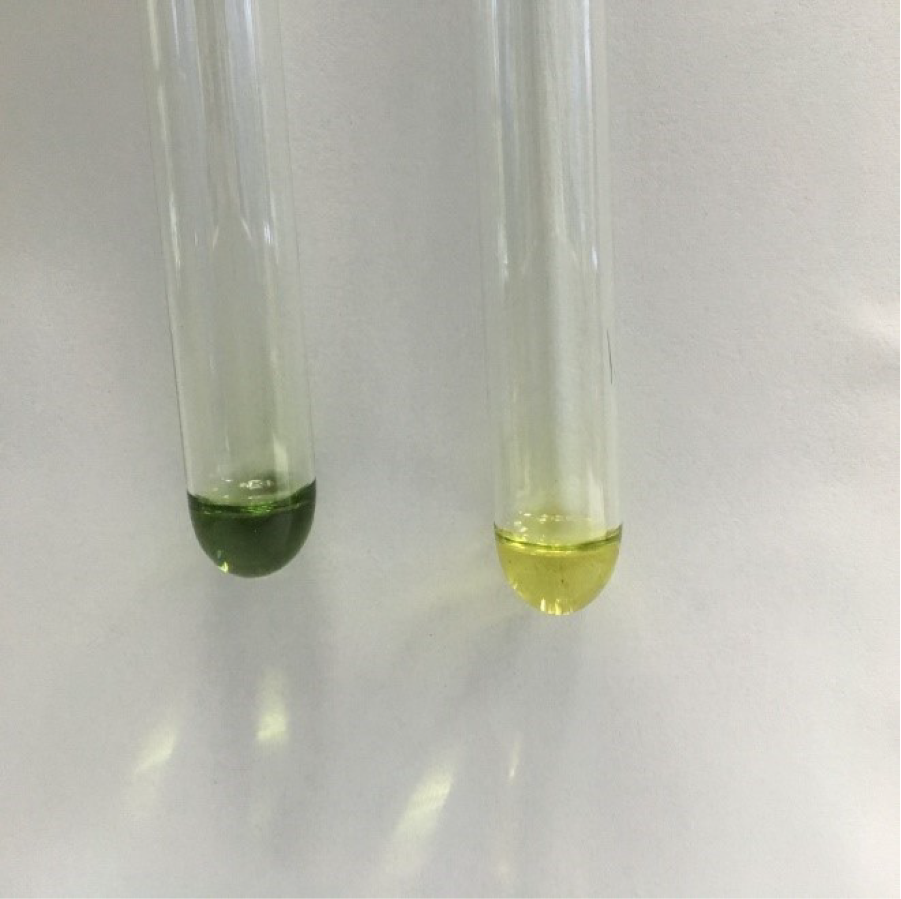

Supplement: S3 Fig — (TIF) [file pone.0190236.s004.tif]
